# Metal-Organic Frameworks to Metal/Metal Oxide Embedded Carbon Matrix: Synthesis, Characterization and Gas Sorption Properties

**DOI:** 10.3390/ma8085245

**Published:** 2015-08-19

**Authors:** Jiun-Jen Chen, Ya-Ting Chen, Duraisamy Senthil Raja, Yu-Hao Kang, Pen-Chang Tseng, Chia-Her Lin

**Affiliations:** 1 Green Energy & Environment Research Laboratories, Industrial Technology Research Institute, Hsinchu 310, Taiwan; E-Mails: JiunJenChen@itri.org.tw (J.-J.C.); derekkang@itri.org.tw (Y.-H.K.); PenChangTseng@itri.org.tw (P.-C.T.); 2 Department of Chemistry, Chung Yuan Christian University, Chung-Li 320, Taiwan; E-Mails: a00510225@hotmail.com (Y.-T.C.); senthilraja1985@gmail.com (D.S.R.); 3 R&D Center for Membrane Technology & Research Center for Structure of Matter, Chung Yuan Christian University, Chung-Li 320, Taiwan

**Keywords:** metal-organic frameworks, 1,4-naphthalene dicarboxylate, nanoporous carbon, metal oxide nanoparticle, gas sorption

## Abstract

Three isostructural metal-organic frameworks, (MOFs), [Fe(OH)(1,4-NDC)] (**1**), [Al(OH)(1,4-NDC)] (**2**), and [In(OH)(1,4-NDC)] (**3**) have been synthesized hydrothermally by using 1,4-naphthalene dicarboxylate (1,4-NDC) as a linker. The MOFs were characterized using various techniques and further used as precursor materials for the synthesis of metal/metal oxide nanoparticles inserted in a carbon matrix through a simple thermal conversion method. The newly synthesized carbon materials were characterized by scanning electron microscopy, transmission electron microscopy, energy-dispersive X-ray spectroscopy analysis, powder X-ray diffraction and BET analysis. The results showed that the MOF-derived carbon composite materials maintained the morphology of the original MOF upon carbonization, and confirmed the insertion of metal/metal oxide particles in the carbon matrix.

## 1. Introduction

Metal/metal oxide nanoparticles have demonstrated distinct properties from their bulk materials and have shown promising applications in many fields such as catalysis [1], magnetism [2] and biochemistry [3]. Though extensive research has been done, the preparation of these nanomaterials still suffers from the aggregation problem. In order to resolve the aggregation issue, a new strategy has been developed in which these nanoparticles are dispersed into carbon matrices such as porous carbon, carbon nanotubes and graphenes [1,2,3]. The carbon matrices can not only block the nanoparticles from aggregation, but also modify their properties [1,2,3,4].

On the other hand, metal-organic frameworks (MOFs) are the new types of porous hybrid functional materials that are constructed by metal ions or metal clusters and organic linkers. Because of their high porosities and tunable structural properties, they have wide functional applications in terms of catalysis [5,6], gas storage [7,8], sensors [9], biomedicine [10], and so on. Apart from the use of MOFs as crystalline porous materials, the utilization of MOFs as a precursor for metal/metal oxide nanoparticle-embedded carbon frameworks has been researched actively in recent years [11,12,13,14,15,16]. The subject has become of particular interest for functional applications such as catalysis, gas storage materials, anode materials for lithium-ion batteries, *etc.* [13,14,15,16]. After the report by Q. Xu *et al.* on the carbonization of MOF-5 [11], various methods have been reported to synthesize materials that contain metal/metal oxide nanoparticle-embedded porous carbon matrix from MOF precursors [17,18,19,20]. Since carbon, metal and oxygen atoms are arranged periodically at the atomic level within MOF structures, the MOFs can be converted into metal/metal oxide nanoparticle-embedded carbon frameworks with a certain degree of permanent porosity, which is much useful for the aforementioned applications.

Further, the synthesis of pure porous carbon materials has also been done using MOF as a precursor approach, which needs to remove the metal/metal oxide nanoparticles formed *in situ*. This can be done using many methods such as vaporization at high temperature, washing with an acid solution and so on. However, our aim is to synthesize the dispersed metal/metal oxide nanoparticles in porous carbon matrices without removing the metal particles formed *in situ* during calcination because of the fact that the MOFs are highly ordered materials, and the carbonized MOFs prepared from these MOFs are expected to induce homogeneously dispersed metal/metal oxide nanoparticles in the resulting porous carbon matrices. Furthermore, MOFs with numerous structural topologies and tunable pores will enable us to construct a variety of carbonized MOFs with different degrees of metal/metal oxide nanoparticle-embedded porous carbon matrices for various applications [21]. 

Though a few articles have been published on the synthesis of the dispersed metal/metal oxide nanoparticles in porous carbon matrices from MOFs, the synthesis of metal/metal oxide nanoparticle-embedded porous carbon hybrid functional materials using a simple, one-step thermal conversion is still a challenge. Hence, herein we report three new metal/metal oxide embedded porous carbon hybrid functional materials from three MOFs ([Fe(OH)(1,4-NDC)] (**1**), [Al(OH)(1,4-NDC)] (**2**), and [In(OH)(1,4-NDC)] (**3**)) of 1,4-naphthalene dicarboxylate (1,4-NDC) using a simple, one-step thermal cracking method. Further, the MOFs and their metal/metal oxide embedded carbon hybrid materials were characterized using various physico-chemical techniques, and their gas sorption properties have been studied.

## 2. Experimental Section

All reagents were commercially available and used as received without further purification. In a typical synthesis of the MOFs, the mixture of FeCl_3_ (0.162 g, 1.0 mmol) or Al(NO_3_)_3_·9H_2_O (0.375 g, 1.0 mmol) or In(NO_3_)_3_·*x*H_2_O (0.301 g, 1.0 mmol), H_2_-1,4-NDC (0.108 g, 0.5 mmol), and H_2_O (10 mL) was placed in a 23 mL Teflon autoclave and then heated at 180 °C for 1 day. After filtering off and washing with distilled water, powder samples of MOFs were obtained. **1**: color, white; yield, 0.104 g; elemental analysis, found/calcd.: C, 39.60/40.13; H, 4.16/4.21% for **1**·4H_2_O. **2**: color, white; yield, 0.117 g; elemental analysis, found/calcd.: C, 49.24/49.00; H, 3.67/3.77% for **2**·2H_2_O. **3**: color, white; yield, 0.113 g; elemental analysis, found/calcd.: C, 38.12/38.64; H, 2.65/2.70% for **3**·1.5H_2_O.

Conversely, the synthesis of metal/metal oxide nanoparticles within a porous carbon matrix involves a direct carbonization method using the above synthesized MOFs as precursors. The MOFs (0.200 g) were taken in a silica boat and then placed in a tube furnace and heated from room temperature to 800 °C under N_2_ gas with a heating rate of 5 °C·min^−1^ to carbonize the MOFs. After reaching the target temperature (800 °C), the temperature was maintained at 800 °C for 5 h, then cooled down to room temperature with a cooling rate of 1 °C·min^−1^. The final black-colored powder products were further characterized. 

## 3. Results and Discussion

**Figure 1:**
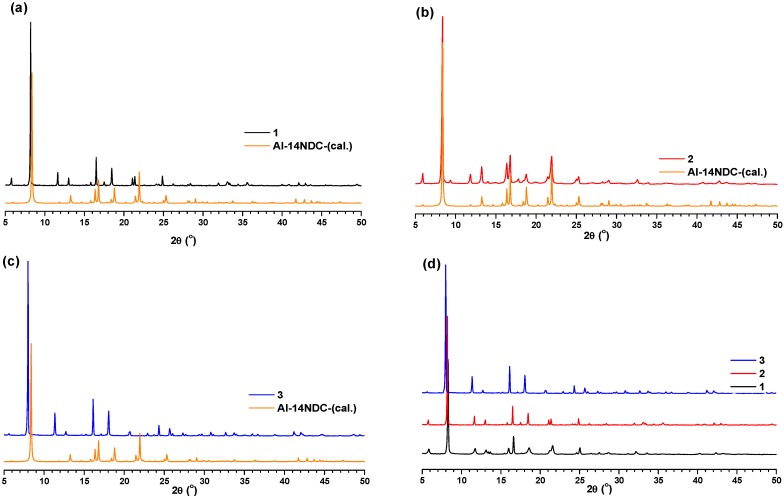
The comparison of PXRD patterns of as-synthesized MOFs, (**a**) **1**, (**b**) **2** and (**c**) **3** with simulated PXRD pattern of reported Al-1,4-NDC; (**d**) the comparison of PXRD patterns of activated MOFs.

The simple hydrothermal reactions of H_2_-1,4-NDC (0.5 mmol) with corresponding metal salts (1.0 mmol) yielded the new MOFs. The new MOFs were initially characterized by elemental analysis, which agrees well with theoretical values. The as-synthesized MOFs may contain guest H_2_O molecules which are removed by heating of these MOFs to 180 °C for 12 h under vacuum (~10^−3^ torr); the phase purity of the bulk materials were independently confirmed by powder X-ray diffraction (PXRD) measurements (Figure 1). Comparing the PXRD patterns of as-synthesized **1**–**3** with calculated PXRD patterns of reported aluminum naphthalenedicarboxylate (Al-1,4-NDC) MOF material [22] confirms that the these new MOFs (**1**–**3**) have the same type of structural architecture as Al-1,4-NDC (Figure 1a–c). Further comparison of these PXRD pattern of activated MOFs with one another showed that the three new MOFs are isostructural to each other (Figure 1d). 

It is to be noted that the diffraction peaks of compound **1** and **3** at 8.2° showed a slight shift from the calculated PXRD of Al-1,4-NDC, which may be due to the difference in the ionic radius of the metal ions (Al^3+^ < Fe^3+^ < In^3+^).

**Figure 2:**
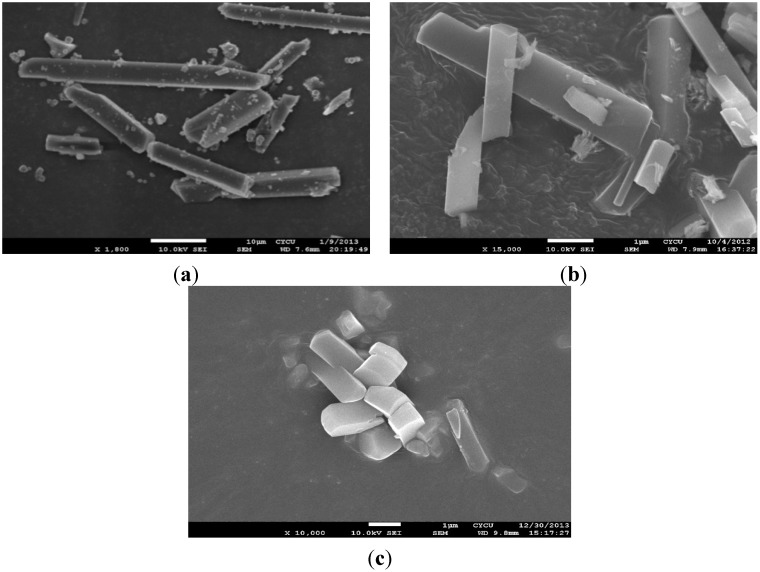
The SEM images of **1** (**a**), **2** (**b**) and **3** (**c**).

The shape and microstructure of the resulting MOFs (**1**–**3**) were investigated via scanning electron microscopy (SEM) and images are presented in Figure 2. The SEM images of the compounds show that they are crystalline materials with an elongated block shape, with individual particle sizes of about 30 μm, 3 μm, and 15 μm (for **1**, **2** and **3**, respectively).

**Figure 3:**
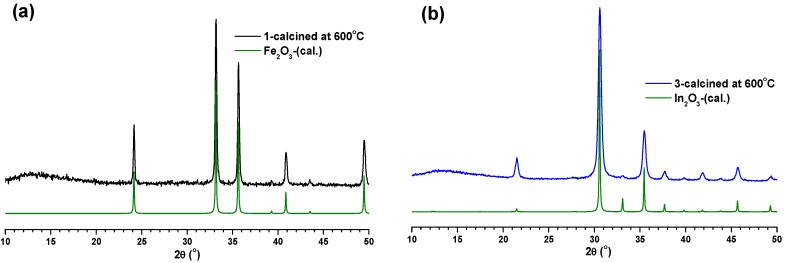
Comparison of PXRD patterns of **1** (**a**) and **3** (**b**) calcined at 600 °C with calculated PXRD pattern of their respective metal oxides.

In order to study the thermal properties of the MOFs, the TGA measurements for the compounds were performed under N_2_ atmosphere, and the results are shown in Figure S1. The slight weight loss observed initially (up 30–150 °C) for all the compounds are may be due to the presence of a small amount of lattice water molecules. TGA studies indicated that **1** and **2** are quite stable up to 300 °C (compound **3** is stable up to 250 °C). These TGA observations have further been confirmed through various *in situ* temperature PXRD measurements for all the compounds (Figures S2–S4). The various temperature PXRD data of Fe-MOF indicated that **1** was converted to Fe_2_O_3_ on heating to temperatures of 350 °C and above (Figure S2), whereas the MOFs **2** and **3** were converted to their respective metal oxides (Al_2_O_3_ and In_2_O_3_) on heating to 400 °C (Figures S3 and S4). Further, the compounds were heated at various temperatures for one hour and the images of the compounds **1**–**3** after calcination at various temperature are given in Figures S5–S7, which show that the compounds did not undergo much color change up to 300 °C due to their high thermal stability. The calcined products of **1**–**3** at 600 °C were characterized using PXRD measurements and compared with calculated PXRD patterns of their corresponding metal oxides. The results indicated that compounds **1** and **3** were converted to their corresponding metal oxides (Fe_2_O_3_ and In_2_O_3_, respectively) during calcination at 600 °C for one hour (Figure 3), whereas the Al-MOF was converted to an unidentified amorphous phase material (Figure S8). 

**Figure 4:**
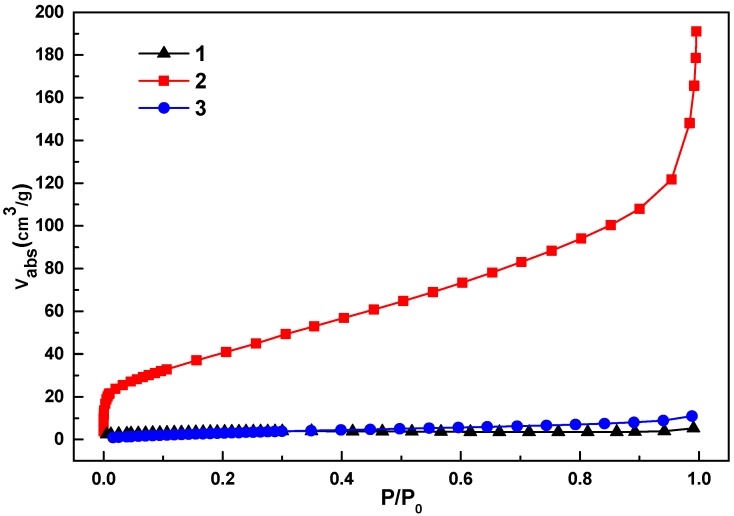
The N_2_ gas adsorption isotherms for **1**–**3** at 77 K.

The porous properties of the MOFs have been analyzed using N_2_ gas sorption measurements at 77K, which are shown in Figure 4. The results showed that compound **2** has a type-I adsorption isotherm which is characteristic of microporous material, and the BET and Langmuir surface area of **2** were calculated to be 155 and 162 m^2^/g, respectively. The N_2_ adsorption isotherms of **1** and **3** indicated the non-porous nature of these materials, which may be due to the bigger ionic radius of the Fe^3+^ and In^3+^ metal ions than that of Al^3+^. However, the CO_2_ gas sorption measurements showed that compounds **1** and **3** have a porous nature.

The CO_2_ gas sorption isotherms for the compounds **1**–**3** at 273 K and 298 K are given in Figures S9–S11. Compounds **1**–**3** exhibited CO_2_ adsorption of 1.30 mmol/g, 2.80 mmol/g, and 2.29 mmol/g, respectively, at 273 K, 1 atm; whereas 0.76 mmol/g, 1.57 mmol/g, and 1.27 mmol/g of CO_2_ adsorption were observed for compounds **1**–**3**, respectively, at 298 K and 1 atm.

**Figure 5:**
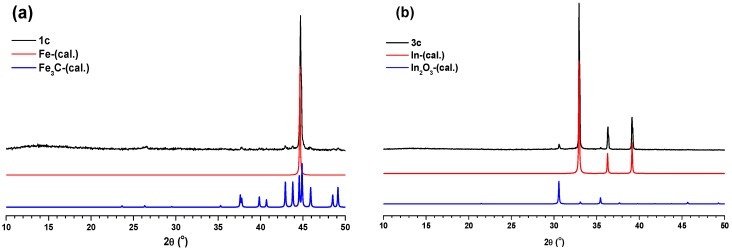
(**a**) Comparison of PXRD pattern of **1c** with calculated PXRD patterns of Fe and Fe_3_C. (**b**) Comparison of PXRD pattern of **3c** with calculated PXRD patterns of In and In_2_O_3_.

In order to prepare metal/metal oxide nanoparticle-embedded carbon materials, the MOFs were used as templates. In a typical carbonization procedure, MOFs (**1**–**3**) were calcined at 800 °C under nitrogen gas flow for 5 h. The final products were characterized with the aid of various techniques. The carbonized products from **1**, **2** and **3** are designated as **1c**, **2c** and **3c**, respectively. Compounds **1c**–**3c** were initially characterized using PXRD measurements. The PXRD pattern matching of **1c** with Fe and Fe_3_C (Figure 5a) clearly indicated the presence of iron and iron carbide particles inside the carbon matrix of **1c**. At the same time, the PXRD pattern of **2c** revealed its amorphous nature (Figure S12). Interestingly, the PXRD pattern matching of **3c** with the theoretical PXRD patterns of In and In_2_O_3_ (Figure 5b) suggested the presence of indium and indium oxide particles inside the carbon matrix of **3c**.

**Figure 6:**
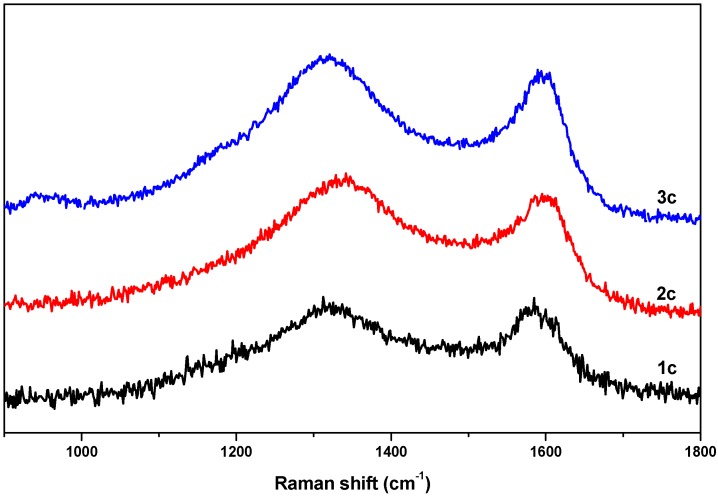
Raman spectra of the obtained nanoporous carbon samples.

Further investigation of the local structure of the synthesized carbon materials (**1c**–**3c**) was carried out via Raman spectroscopy. Raman spectra of the obtained carbon samples are shown in Figure 6, exhibiting D and G bands centered around 1320 cm^−1^ and 1600 cm^−1^, respectively, which is due the disordered carbon structures and the stretching vibrations in opposite directions of two carbon atoms in a graphene sheet. The relative ratios of the G band to the D band (*I_G_*/*I_D_*) were found to be 1.11, 1.15 and 1.18 for **1c**, **2c** and **3c**, respectively, suggesting that in all samples the graphene sheets were not well-developed and the local carbon structures contained both graphitic and disordered carbon atoms [23].

**Figure 7:**
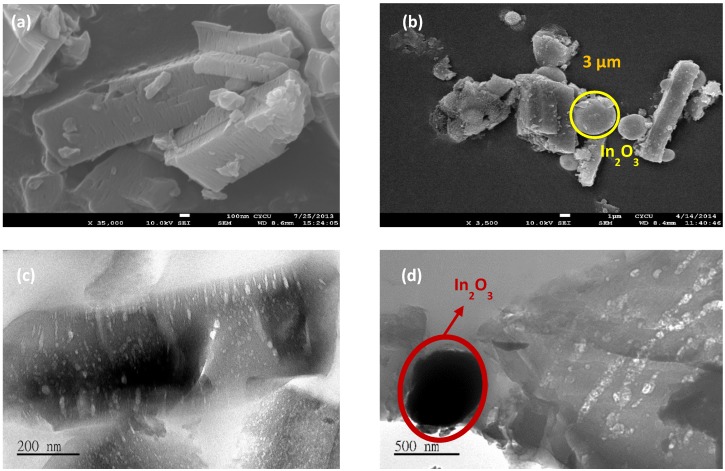
The SEM images of **2c** (**a**), and **3c** (**b**) and the TEM images of **2c** (**c**), and **3c** (**d**).

Since compound **1c** exhibited a strong interaction with a magnetic field (Figure S13), we were unable to characterize it further using SEM, TEM or energy dispersive X-ray spectroscopy (EDS) analysis. The SEM and TEM images of **2c** and **3c** (Figure 7) suggested that the obtained nanoporous carbons retained a typical crystal morphology similar to that of the parent MOFs. Further, the SEM and TEM images of **3c** revealed that the surface of nanoporous carbon samples was embedded with indium oxide nanoparticles (Figure 3b,d). 

**Figure 8:**
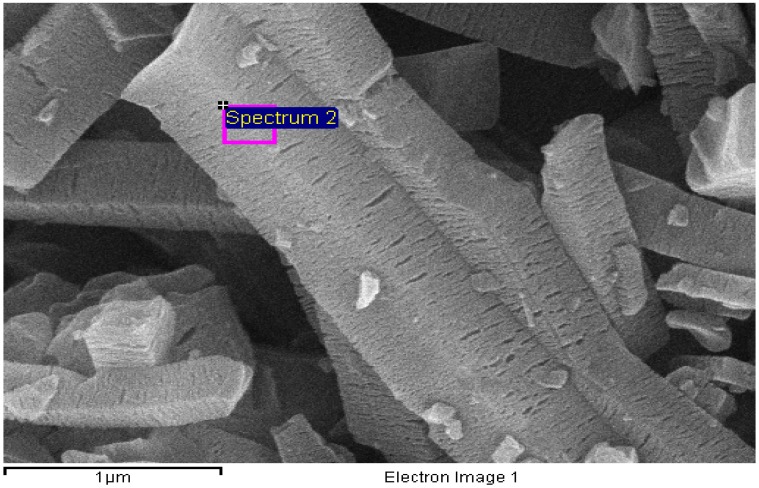

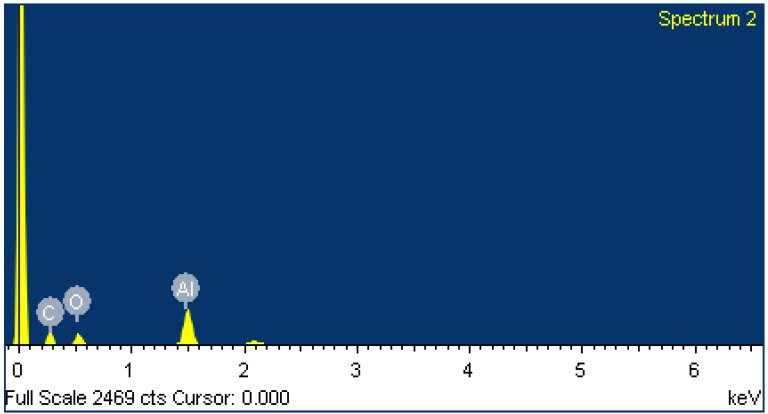
The EDS analysis results for **2c**.

The composition of the carbon matrix and the metal oxide nanoparticles were confirmed through EDS analysis, and the results are given in Figure 8 and Figure S14. Surprisingly, the EDS analysis of **2c** showed the presence of aluminum oxide nanoparticles inside the carbon matrix (Figure 8). On the other hand, the results of EDS analysis of **3c** at two different SEM image locations clearly confirms the presence of In_2_O_3_ in the carbon framework of **3c** (Figure S14).

**Table:** 

Element	Weight (%)	Atomic (%)
C	45.36	58.85
O	24.22	23.59
Al	30.42	17.57

**Figure 9:**
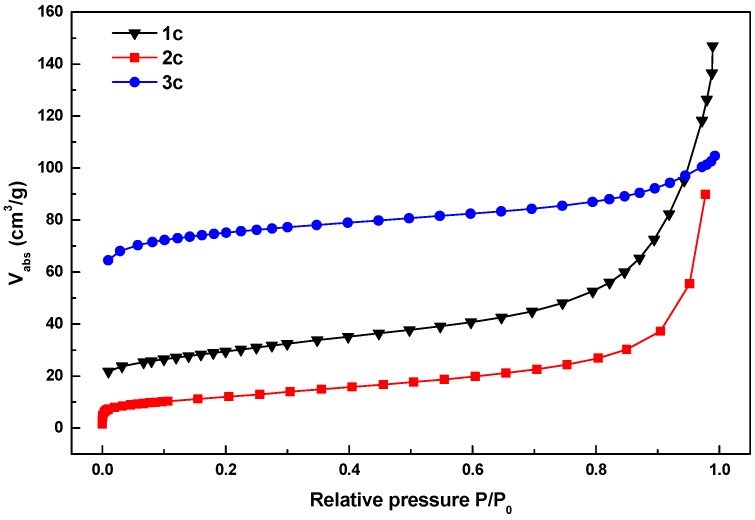
The N_2_ gas adsorption isotherms for **1c**–**3c** at 77 K.

Nitrogen gas adsorption analysis was used to further characterize the nanoporous structure of **1c**, **2c** and **3c** (Figure 9). The shape of the isotherms indicated the existence of both micropores and mesopores. The steep increase at low relative pressure indicates the presence of the micropores. The isotherms showed a small hysteresis, which is typical for the presence of spherical mesopores randomly connected with weak microporosity. The analyzed pore characteristics of the carbon materials are summarized in Table 1. It is interesting to note that the porous properties of the carbon materials are better than that of their parent MOF materials. This may be due to the fact that some of the linker units of the MOFs have been removed in the form of CO_2_ gas during the carbonization process, which makes the resulting carbon materials more porous than the parent MOF materials. 

**Table 1:** Pore characteristics of the synthesized nanoporous carbon materials.

Materials	BET Surface Area (m^2^/g)	Langmuir Surface Area (m^2^/g)	Total Pore Volume (cm^3^/g) P/P_o_~0.99
**1c**	155	261	0.28
**2c**	100	149	0.21
**3c**	236	342	0.16

## 4. Conclusions

In summary, Fe-, Al-, and In-MOFs based on a 1,4-NDC linker have been prepared and characterized. Using these MOFs as precursor materials, metal/metal oxide nanoparticle-inserted nanoporous carbons were prepared via simple direct carbonization without using any additional carbon sources. The PXRD, SEM, TEM, EDS and nitrogen sorption measurements confirm the dispersion of metal/metal oxide nanoparticles in the resulting nanoporous carbon materials. It can be expected that these newly synthesized carbon composite materials have promising prospects for application in supercapacitors.

## Supplementary Materials

Supplementary materials can be accessed at: http://www.mdpi.com/1996-1944/8/8/5336/s1.
