# Supplementary material for: Metal-Organic Frameworks to Metal/Metal Oxide Embedded Carbon Matrix: Synthesis, Characterization and Gas Sorption Properties
Source: Materials (Basel). 2015 Aug 19;8(8):5336–47. doi: 10.3390/ma8085245 (PMC5455493; doi:10.3390/ma8085245)
Supplement: Supplementary file 1 [file materials-08-05245-s001.pdf]

Supplementary Materials

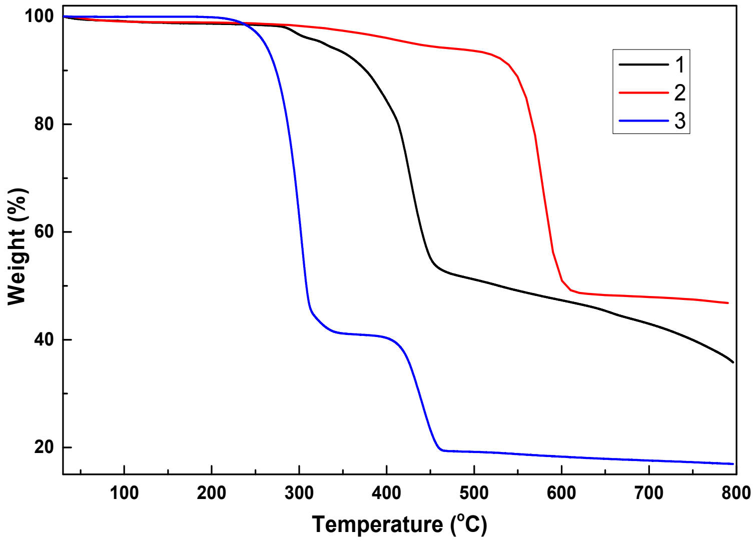

Figure S1. TGA for compounds **1–3**.

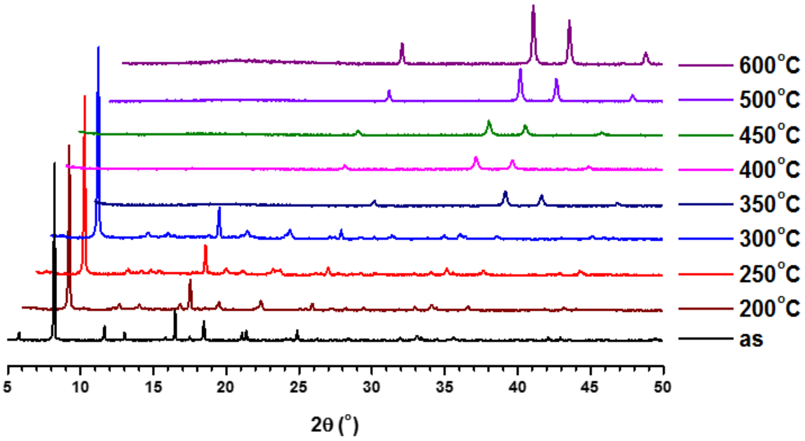

Figure S2. Varied temperatures PXRD patterns of **1**.

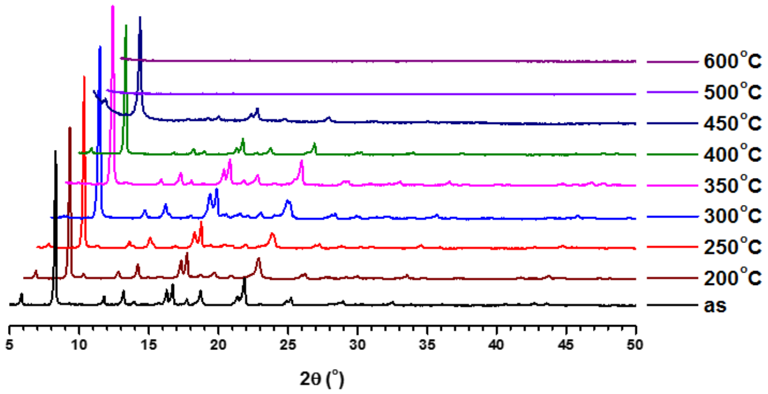

Figure S3. Varied temperatures PXRD patterns of **2**.

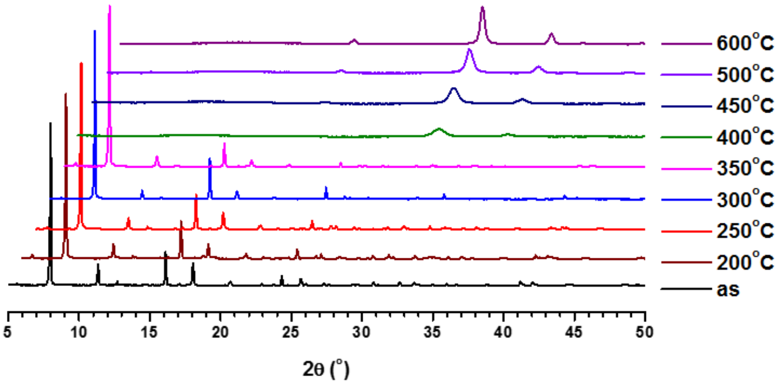

Figure S4. Varied temperatures PXRD patterns of **3**.

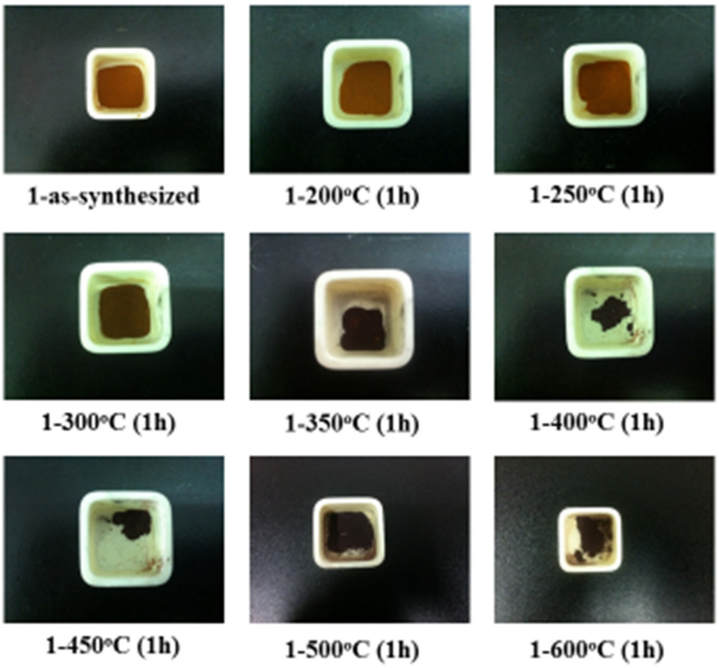

Figure S5. Photos of **1** after calcination at various temperatures for one hour.

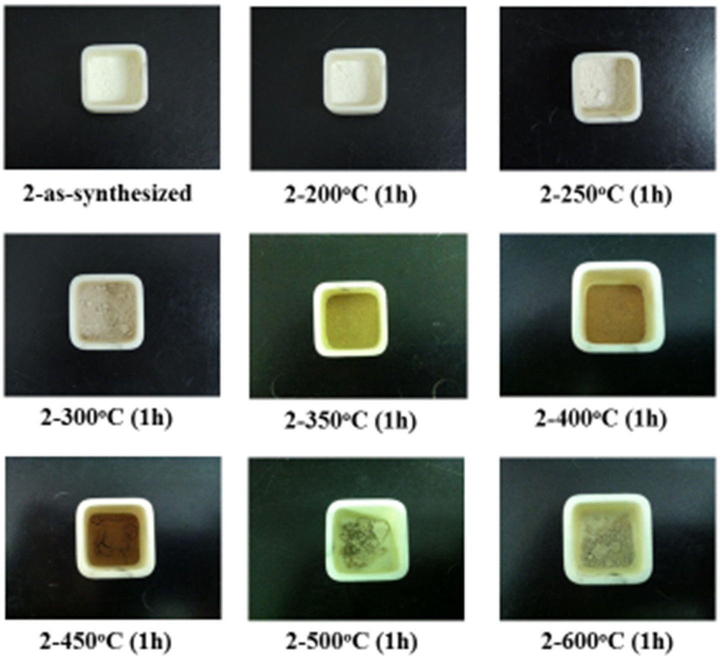

Figure S6. Photos of **2** after calcination at various temperatures for one hour.

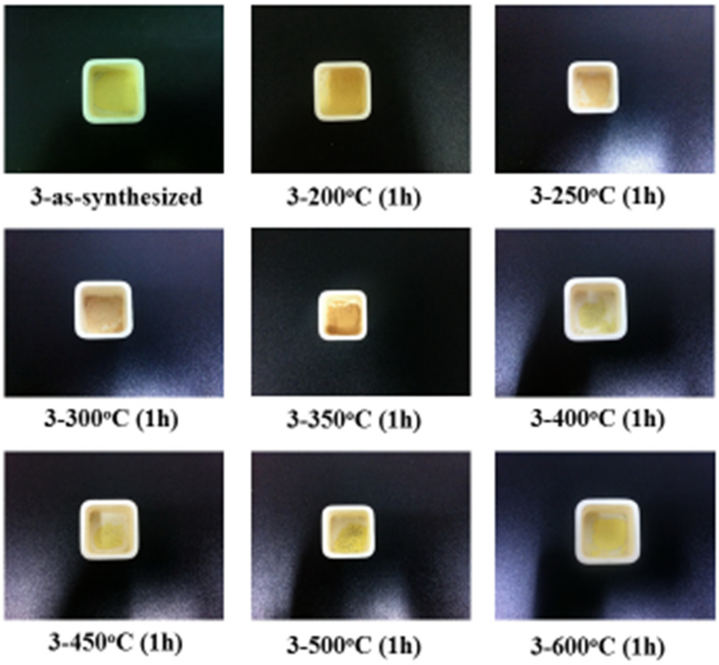

Figure S7. Photos of **3** after calcination at various temperatures for one hour.

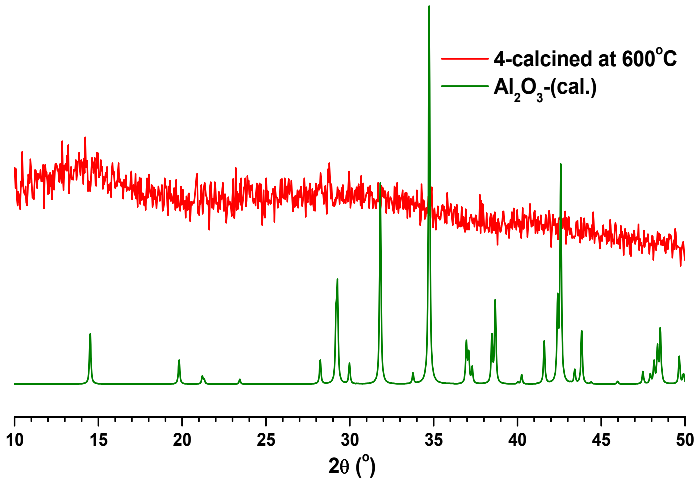

Figure S8. Comparison of PXRD pattern of **2** calcined at 600 °C with calculated PXRD pattern of Al_2O_3.

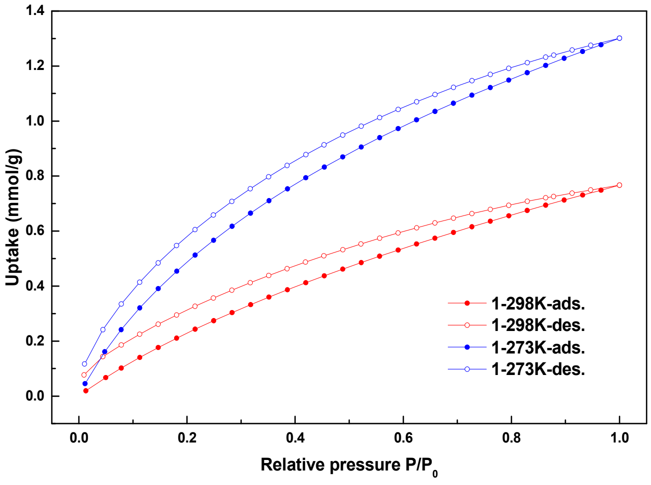

Figure S9. The CO_2 gas sorption isotherms for **1** at 273 K and 298 K.

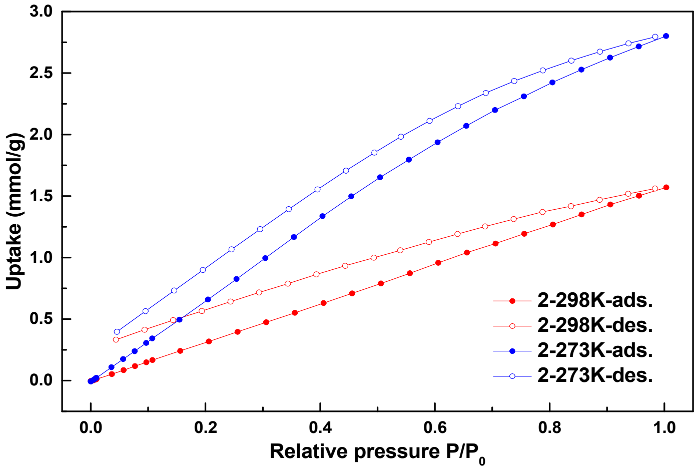

Figure S10. The CO_2 gas sorption isotherms for **2** at 273 K and 298 K.

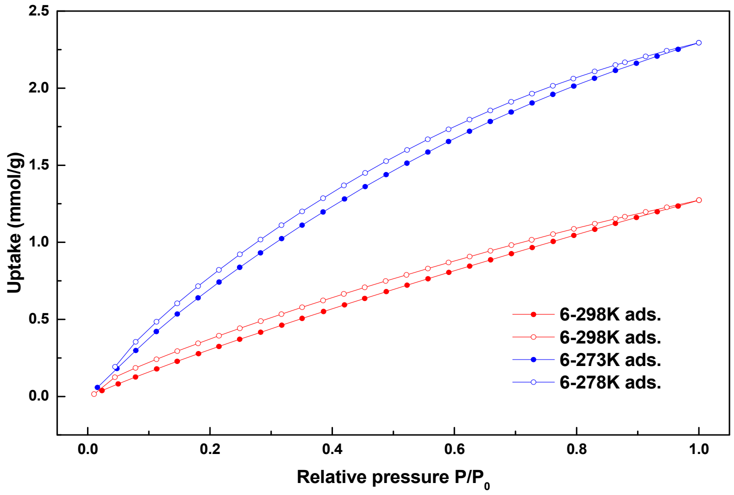

Figure S11. The CO_2 gas sorption isotherms for **3** at 273 K and 298 K.

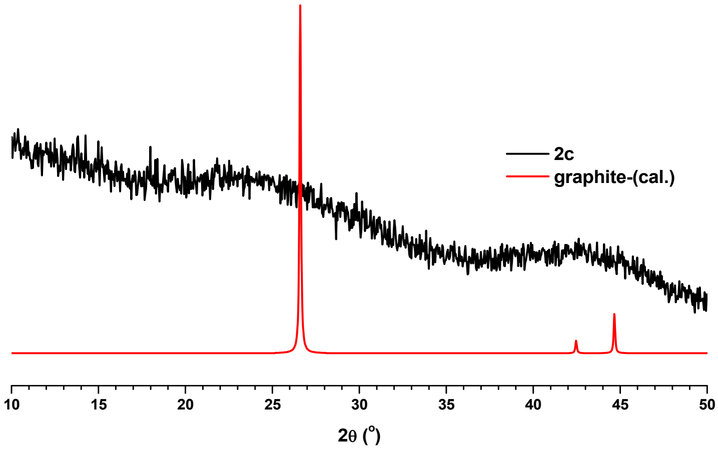

Figure S12. Comparison of PXRD pattern of **2c** with calculated PXRD pattern of Graphite.

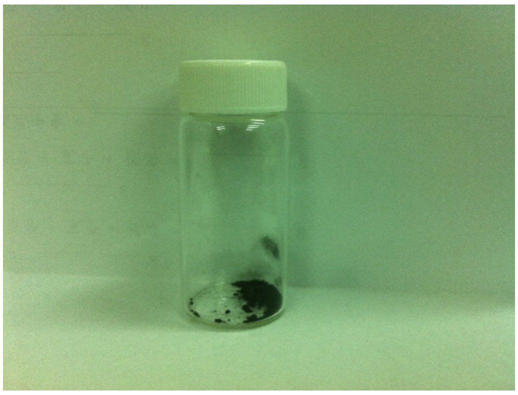

(**a**)

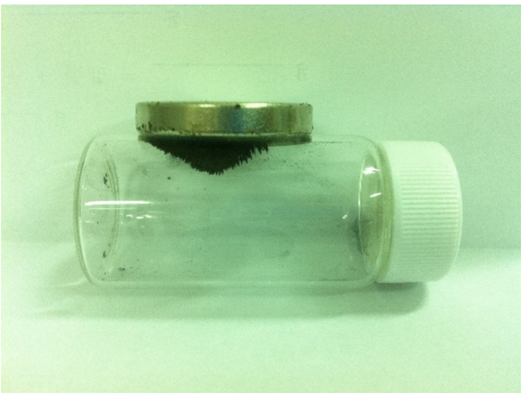

(**b**)

Figure S13. Photos of **1c** without magnetic field (**a**) and with magnetic field (**b**).

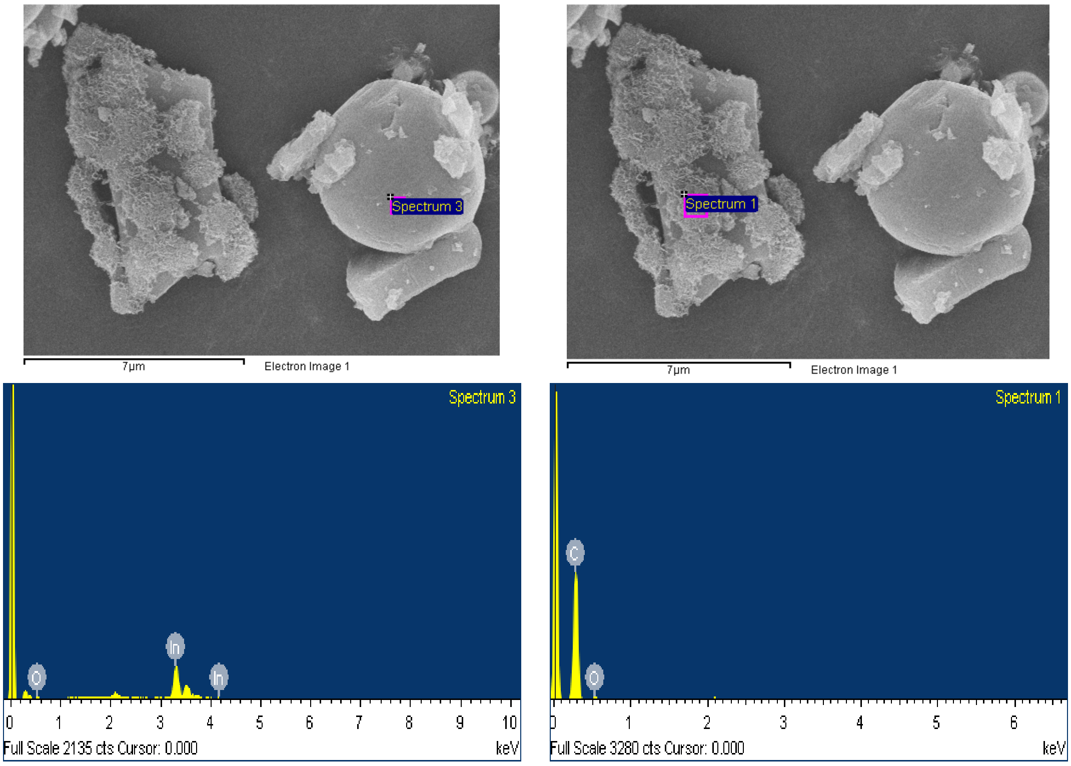

(**a**) (**b**)

Element	Weight (%)	Atomic (%)	Element	Weight (%)	Atomic (%)
C	93.22	94.83	C	0.00	0.00
O	6.78	5.17	O	4.36	24.67
In	0.00	0.00	In	95.64	75.33

Figure S14. The EDS analysis results for **3c** at two different places of SEM images (**a**) and (**b**).
